# Comment on ‘Bidirectional Transitions of Sarcopenia States in Older Adults: The Longitudinal Evidence From CHARLS’ by Luo et al.

**DOI:** 10.1002/jcsm.13593

**Published:** 2024-09-30

**Authors:** Huanhuan Feng, Han Wang, Wenchao Zhou

**Affiliations:** ^1^ Orthopaedics Department Taicang TCM Hospital Affiliated to Nanjing University of Chinese Medicine Suzhou China; ^2^ Acupuncture and Massage Department Affiliated Sports Hospital of Chengdu Sport University Chengdu China

To the Editor

We are writing in response to the article ‘Bidirectional Transitions of Sarcopenia States in Older Adults: The Longitudinal Evidence from CHARLS’ [1]. This study significantly advances our understanding of the complex relationships between the probability and intensity of transition from non‐sarcopenia to possible sarcopenia, sarcopenia and death in older adults. It also highlights the critical role that early screening and intervention can play in preventing the progression of sarcopenia. We commend the authors for their valuable contributions and offer several suggestions for further consideration.

First, this study primarily focuses on the elderly population in China, lacking direct comparisons with other countries or regions. As a result, the findings may not be generalizable to older adults in different cultural contexts or healthcare systems, highlighting a clear regional limitation. Second, although the study utilized a multivariate Markov model (MSM) to analyse transition probabilities in subgroups such as age, body mass index (BMI) and physical function impairment, further subgroup analyses are recommended. These could include examining sex differences, mental health status, lifestyle factors, comorbid chronic conditions and socioeconomic status to better understand their impact on sarcopenia transitions in older adults [2, 3, 4]. Such analyses would enable the development of more targeted and personalized intervention strategies. Third, although the study analysed transitions over different years, the follow‐up period was relatively short, averaging 3.29 years. Given that sarcopenia is a chronic and progressive condition, longer follow‐up periods could yield more comprehensive data and more robust analytical results.

As healthcare professionals, we recognize a significant opportunity to contribute to this field. Although the study did not specifically address early intervention strategies, we can effectively slow the progression of sarcopenia and improve the physical function and quality of life in older adults through early interventions such as regular screening and monitoring, personalized exercise programmes, nutritional support, lifestyle modifications, management of comorbidities and providing psychological support and social engagement.

In conclusion, we greatly appreciate the valuable insights this article provides on sarcopenia outcomes. Building on this research, we can develop more targeted intervention strategies for managing sarcopenia in older adults.

## Conflicts of Interest

The authors declare that the research was conducted in the absence of any commercial or financial relationships that could be construed as a potential conflict of interest.
